# High prevalence and predominance of *BRCA1* germline mutations in Pakistani triple-negative breast cancer patients

**DOI:** 10.1186/s12885-016-2698-y

**Published:** 2016-08-23

**Authors:** Muhammad Usman Rashid, Noor Muhammad, Seerat Bajwa, Saima Faisal, Muhammad Tahseen, Justo Lorenzo Bermejo, Asim Amin, Asif Loya, Ute Hamann

**Affiliations:** 1 Shaukat Khanum Memorial Cancer Hospital and Research Centre (SKMCH & RC), Lahore, Pakistan; 2 German Cancer Research Center (DKFZ), Molecular Genetics of Breast Cancer, Heidelberg, Germany; 3 Institute of Medical Biometry and Informatics, University of Heidelberg, Heidelberg, Germany; 4 Levine Cancer Institute (LCI), Charlotte, USA

**Keywords:** *BRCA1/2*, Breast cancer, Germline mutations, Pakistan, Triple-negative breast cancer

## Abstract

**Background:**

Women harboring *BRCA1/2* germline mutations have high lifetime risk of developing breast/ovarian cancer. The recommendation to pursue *BRCA1/2* testing is based on patient’s family history of breast/ovarian cancer, age of disease-onset and/or pathologic parameters of breast tumors. Here, we investigated if diagnosis of triple-negative breast cancer (TNBC) independently increases risk of carrying a *BRCA1/2* mutation in Pakistan.

**Methods:**

Five hundred and twenty-three breast cancer patients including 237 diagnosed ≤ 30 years of age and 286 with a family history of breast/ovarian cancer were screened for *BRCA1/2* small-range mutations and large genomic rearrangements. Immunohistochemical analyses were performed at one center. Univariate and multiple logistic regression models were used to investigate possible differences in prevalence of *BRCA1/2* mutations according to patient and tumor characteristics.

**Results:**

Thirty-seven percent of patients presented with TNBC. The prevalence of *BRCA1* mutations was higher in patients with TNBC than non-TNBC (37 % *vs.* 10 %, *P* < 0.0001). 1 % of TNBC patients were observed to have *BRCA2* mutations*.* Subgroup analyses revealed a larger proportion of *BRCA1* mutations in TNBC than non-TNBC among patients 1) diagnosed at early-age with no family history of breast/ovarian cancer (14 % *vs.* 5 %, *P* = 0.03), 2) diagnosed at early-age irrespective of family history (28 % *vs*. 11 %, *P* = 0.0003), 3) had a family history of breast cancer (49 % *vs.* 12 %, *P* < 0.0001), and 4) those with family history of breast and ovarian cancer (81 % *vs.* 28 %, *P* = 0.0005). TNBC patients harboring *BRCA1* mutations were diagnosed at a later age than non-carriers (median age at diagnosis: 30 years (range 22–53) *vs*. 28 years (range 18–67), *P* = 0.002). The association between TNBC status and presence of *BRCA1* mutations was independent of the simultaneous consideration of family phenotype, tumor histology and grade in a multiple logistic regression model (Ratio of the probability of carrying *BRCA1/2* mutations for TNBC *vs.* non-TNBC 4.23; 95 % CI 2.50–7.14; *P* < 0.0001).

**Conclusion:**

Genetic *BRCA1* testing should be considered for Pakistani women diagnosed with TNBC.

**Electronic supplementary material:**

The online version of this article (doi:10.1186/s12885-016-2698-y) contains supplementary material, which is available to authorized users.

## Background

Women carrying a pathogenic germline mutation in the *BRCA1* and *BRCA2* genes have an increased lifetime risk of developing breast, ovarian, and several other cancers [1]. The identification of women harboring mutations in these genes is clinically important and has a significant socio-cultural impact. A major challenge faced by physicians is to identify most appropriate candidates for genetic *BRCA1/2* testing since the cost of comprehensive genetic testing can be high and only 3 % of all breast cancers are attributed to *BRCA1/2* germline mutations.

The decision to offer genetic testing to a breast cancer patient is currently based on family history of breast/ovarian cancer and age of disease onset. Several prediction models, which consider age of onset and family history of cancer, can be used to estimate the prior probability of having a *BRCA1* or *BRCA2* mutation [2]. In addition, histopathological tumor parameters can be considered to help predict the presence of a mutation.

Triple negative breast cancer (TNBC) is defined by the absence of estrogen receptor (ER), progesterone receptor (PR), human epidermal growth factor receptor 2 (HER2) and accounts for 12–15 % of all invasive breast cancer [3]. It occurs most frequently in young women and African-Americans. In Pakistan, 10-year outcome analysis of 636 breast cancer patients registered at a tertiary-care cancer center (Shaukat Khanum Memorial Cancer Hospital and Research Centre - SKMCH & RC) showed that 30.5 % (194/636) of the cases had TNBC; and majority (56.2 %) had their diagnosis made at less than 40 years of age [4]. Patients with TNBC are known to have unfavorable survival compared to patients with other breast cancer subtypes [5].

A large proportion of tumors in women with *BRCA1* mutations are associated with the TNBC phenotype [6]. *BRCA1/2* mutations have been identified with frequencies varying from 9.4 to 15.4 % in unselected, 17.4 to 49.1 % in younger age and 11.6 to 62 % in high risk patients with TNBC [7–15]. Studies reporting the frequency of *BRCA1/2* mutations in TNBC patients from Asia have had several deficiencies including small population size [16–18], restriction of analysis to *BRCA1* gene [19, 20] and evaluation limited to small-range mutations [16, 21, 22]. In order to determine the utility of genetic testing for *BRCA1* and *BRCA2* germline mutations for women with TNBC in Pakistan, we comprehensively screened both genes for small-range mutations as well as large genomic rearrangements in a group of 523 breast cancer patients who were selected based on early-age of disease onset or family history of breast/ovarian cancer, including 192 patients diagnosed with TNBC.

## Methods

### Study subjects

**Fig. 1:**
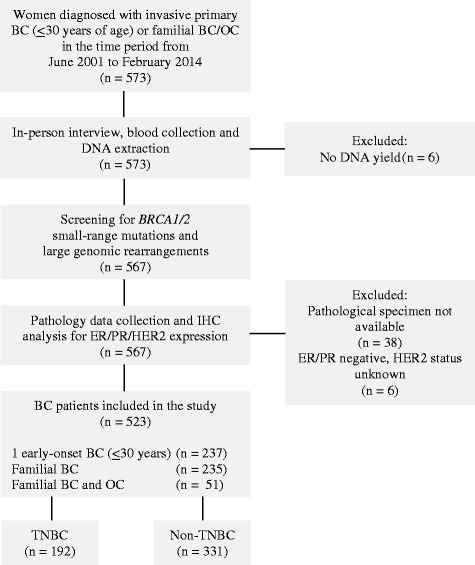
Description of the study participants. BC, breast cancer; ER, estrogen receptor; HER2, human epidermal growth factor receptor 2; OC, ovarian cancer; PR, progesterone receptor; TNBC, triple-negative breast cancer

Index patients included in this study had a diagnosis of primary invasive breast cancer and were selected based on the following criteria: 1) one female breast cancer diagnosed ≤ 30 years of age; 2) two or more first- or second-degree (through a male) female relatives diagnosed with breast cancer with at least one diagnosed ≤ 50 years of age; or 3) at least one female breast cancer and one ovarian cancer at any age. A total of 573 women recruited at the SKMCH & RC in Lahore, Pakistan, from June 2001 to February 2014 fulfilled these criteria. Blood samples were obtained from all patients for the isolation of genomic DNA. Clinical, histopathologic and risk factor data were collected from all study participants. Fifty patients were excluded from the study. Reasons for exclusion are detailed in Fig. 1.

The study was approved by the Ethical Review Board of the SKMCH & RC. All study participants signed informed written consent.

### *BRCA1/2* screening for small-range mutations and large genomic rearrangements

Genomic DNA was isolated as previously described [23]. One hundred and twenty-one cases comprehensively screened for *BRCA1* (Genbank accession number U14680.1) and *BRCA2* (Genbank accession number U43746.1) small-range mutations using protein-truncation test (PTT), single-strand conformational polymorphism analysis (SSCP) and denaturing high-performance liquid chromatography (DHPLC) analysis followed by DNA sequencing of variant fragments, and 26 *BRCA1/2* mutations was described in an earlier report [23] (primer sequences are available upon request). When available, a mutation positive control was included in each set of PTT, SSCP and DHPLC analyses. A description of the *BRCA1/2* screening methods is given in Supplementary methods (Additional file 1). The remaining 402 cases recruited subsequently were screened for *BRCA1/2* small-range mutations using DHPLC and DNA sequencing analyses. Of these, 295 cases were previously described [24]. All patients negative for small-range *BRCA1/2* mutations were further screened for large genomic rearrangements. Multiplex ligation-dependent probe amplification (MLPA) analysis was performed using probe mix P003 and P087 for *BRCA1* and probe mix P045 for *BRCA2* according to the manufacturer’s instructions (MRC Holland, Amsterdam, The Netherlands).

### Immunohistochemical (IHC) analysis

Formalin-fixed paraffin-embedded (FFPE) blocks were retrieved from the pathology department; blocks were not available for 38 patients (Fig. 1). Tumor grade was assigned using the Nottingham Histologic Score. IHC analysis of ER, PR and HER2 expression was performed using standard methods [25]. Slides were interpreted by a trained breast pathologist who was blinded to *BRCA1/2* mutation status. Tumors were considered negative for ER and PR if < 1 % of tumor cells demonstrated positive nuclear staining. Tumors were considered negative for HER2 if IHC score was 0 or 1+. Cases with IHC score 2+ were further subjected to fluorescence *in situ* hybridization (FISH) using the PathVysion® HER2 DNA probe kit (Abbott Laboratories, Abbott Park, IL). Tumors with a HER2/CEP17 ratio of > 2.2 and tumors with IHC score 3+ were considered positive.

### Statistical analysis

The comparison of the distribution of clinical and histopathological characteristics between *BRCA1/2* carriers and non-carriers was done using Fisher’s exact test for categorical variables and the Wilcoxon rank-sum test for quantitative variables. Univariate and multiple logistic regression models were used to investigate possible differences in the prevalence of *BRCA1/2* mutations according to patient and tumor characteristics. All statistical tests were two sided. Results were deemed statistically significant if the *P* value was 0.05 or less. All statistical computations were done using StatXact 4 for Windows (Cytel Inc., Cambridge, USA), SAS version 9.3 and R, version 2.1.

## Results

### Clinical characteristics of the study participants and histopathologic parameters of tumors according to TNBC status

In total 523 unrelated Pakistani women diagnosed with primary invasive breast cancer were included in the study. Of these, 45.3 % were diagnosed at young age (≤ 30 years) and 54.7 % reported a positive family history of breast/ovarian cancer. IHC analysis of ER, PR, and HER2 expression showed that 36.7 % of the patients presented with TNBC. Compared to non-TNBC patients, women with TNBC had an earlier age of diagnosis (31.6 years (range 18–67) and 35.6 years (range 19–73), respectively; *P* < 0.0001, Wilcoxon rank-sum test), were more often premenopausal (91.1 % *vs*. 80.7 %, *P* = 0.002) and of Punjabi ethnicity (79.5 % *vs*. 69.2 %, *P* = 0.03). TNBC tumors were observed to have greater propensity for invasive ductal carcinoma compared to non-TNBC (96.4 % *vs*. 89.4 %, *P* = 0.004), higher tumor grade 3 (88.8 % *vs*. 62.9 %, *P* < 0.0001), and lymph node negativity (53.9 % *vs*. 32.5 %, *P* < 0.0001). Selected clinical and histopathologic characteristics of the study participants by TNBC status are shown in Table 1.

**Table 1:** Selected clinical and pathological characteristics of the 523 Pakistani cases according to TNBC status

Parameters	TNBC (*N* = 192)	Non-TNBC (*N* = 331)	*P* ^a^
	n (%)	n (%)	
Age at diagnosis of BC (years)			
Mean	31.6	35.6	**< 0.0001** ^b^
Range	18–67	19–73	
Family phenotype			
1 early-onset BC (≤30 years)	90 (46.9)	147 (44.4)	NS^c^
Familial BC	76 (39.6)	159 (48.0)	
Familial BC and OC	26 (13.6)	25 (7.6)	
Menopausal status			
Premenopausal	174 (91.1)	267 (80.7)	**0.002** ^d^
Postmenopausal	17 (8.9)	64 (19.3)	
Unknown	1	0	
Ethnicity			
Punjabi	151 (79.5)	229 (69.2)	**0.03** ^e^
Pathan	19 (10.0)	54 (16.3)	
Others	20 (10.5)	48 (14.5)	
Unknown	2	0	
Histology			
Ductal	185 (96.4)	294 (89.4)	**0.004** ^f^
Lobular	1 (0.5)	15 (4.6)	
Mixed^g^	2 (1.0)	11 (3.3)	
Mucinous	0	6 (1.8)	
Metaplastic	2 (1.0)	3 (0.9)	
Medullary	2 (1.0)	0	
Unknown	0	2	
Tumor size			
pT1	30 (20.3)	71 (26.9)	NS^h^
pT2	91 (61.5)	147 (55.7)	
pT3	24 (16.2)	44 (16.7)	
pT4	3 (2.0)	2 (0.7)	
Unknown	44	67	
Tumor grade (Nottingham)			
1	0	5 (1.6)	**< 0.0001** ^i^
2	20 (11.2)	111 (35.5)	
3	158 (88.8)	197 (62.9)	
Unknown	14	18	
Lymph node status			
Positive	83 (46.1)	212 (67.5)	**< 0.0001** ^j^
Negative	97 (53.9)	102 (32.5)	
Unknown	12	17	

*P* values marked in bold are statistically significant

*BC* breast cancer, *NS* non-significant, *OC* ovarian cancer, *TNBC* triple-negative breast cancer. ^a^Fisher’s exact test. ^b^Wilcoxon rank-sum test. ^c^Early-onset *vs.* familial BC and early-onset *vs.* familial BC and OC. ^d^Premenopausal *vs.* postmenopausal. ^e^Punjabi *vs.* Pathan. ^f^Ductal *vs.* others. ^g^Including ductal carcinomas with lobular and mucinous features. ^h^pT1 *vs.* pT2+. ^i^Grade 1, 2 *vs.* 3. ^j^Lymph node positive *vs*. negative

### *BRCA1/2* mutation prevalence in patients with TNBC and non-TNBC

The complete coding regions of the *BRCA1* and *BRCA2* genes were screened for small-range mutations and large genomic rearrangements in all 523 breast cancer patients. Overall, 125 cases with deleterious mutations were identified, of these, 105 occurred in *BRCA1* (84 %) and 20 (16 %) in *BRCA2* (Table 2). *BRCA1* mutations were more frequent in patients with TNBC than in those with non-TNBC (37 % *vs.* 10.3 %, *P* < 0.0001). Majority of the mutations in patients with TNBC, 97.3 % (71/73), were detected in *BRCA1*; 2.7 % (2/73) had mutations in *BRCA2* (*P* < 0.0001) (Additional file 2: Table S1). The corresponding percentage for *BRCA1* and *BRCA2* mutations in non-TNBC cases was 65.4 % (34/52) and 34.6 % (18/52) (*P* = 0.04).

**Table 2:** *BRCA1/2* mutation frequencies in patients with TNBC and non-TNBC

	TNBC (*N* = 192)	Non-TNBC (*N* = 331)	*P* ^a^
Mutation status	*n* (%)	*n* (%)	
Carriers	73 (38.0)	52 (15.7)	**< 0.0001** ^b^
*BRCA1*	71 (37.0)	34 (10.3)	**< 0.0001** ^c^
*BRCA2*	2 (1.0)	18 (5.4)	NS^d^
Non-carriers	119 (62.0)	279 (84.3)	

*P* values marked in bold are statistically significant

*NS* non-significant, *TNBC* triple-negative breast cancer

^a^Fisher’s exact test. ^b^Carriers *vs.* non-carriers. ^c^ *BRCA1* carriers *vs.* non-carriers

^d^ *BRCA2* carriers *vs.* non-carriers

In this study, patients with TNBC harboring a *BRCA1* mutation (*n* = 71) were older than *BRCA1/2* non-carriers (*n* = 119) with mean age of diagnosis 32.9 years (range 22–53) and 30.9 years (range 18–67), respectively (*P* = 0.002, Exact Wilcoxon rank-sum test). The mean age for non-TNBC patients was 33.5 years (range 21–72) for *BRCA1* carriers (*n* = 34), 36.8 (range 25–54) for *BRCA2* carriers (*n* = 18) and 35.7 (range 19–73) for non-carriers (*n* = 279).

### Subgroup analysis by family phenotype, age of diagnosis, and ethnicity

Prevalence of *BRCA1/2* mutations in patients with TNBC and non-TNBC distributed by family phenotype, age of diagnosis, and ethnicity is detailed in Table 3. Among patients with TNBC, *BRCA1* mutations were identified in 14.4 % of patients with early-onset disease (≤ 30 years), 48.7 % of patients with familial breast cancer and in 80.8 % of patients with familial breast and ovarian cancer. These frequencies were higher than the corresponding frequencies of 5.4, 12.0 and 28.0 % observed in non-TNBC patients (*P* = 0.03, *P* < 0.0001 and *P* = 0.0005, respectively). Mutations in *BRCA2* were detected in 2.6 % of patients with familial breast cancer and were absent in those with early-onset disease and familial breast and ovarian cancer. Similar frequencies were observed in non-TNBC patients; 4.1 % in patients with early-onset disease, 6.9 % in those with familial breast cancer, and 4.0 % in patients with familial breast and ovarian cancer (*P* = 0.09, *P =* 0.73 and *P* = 1.0, respectively).

**Table 3:** *BRCA1/2* mutation frequencies in patients with TNBC and non-TNBC, by age-at-diagnosis, family phenotype and ethnicity

Variables	TNBC (*N* = 192)					Non-TNBC (*N* = 331)					*P* ^a^
	No. of cases	No. of mutations (%) in			Non-	No. of cases	No. of mutations (%) in			Non-	
		*BRCA1*	*BRCA2*	*BRCA1/2*	carriers		*BRCA1*	*BRCA2*	*BRCA1/2*	carriers	
Family phenotype											
1 early-onset BC (≤ 30 years)	90	13 (14.4)	0 (0)	13 (14.4)	77	147	8 (5.4)	6 (4.1)	14 (9.5)	133	**0.03**
Familial BC	76	37 (48.7)	2 (2.6)	39 (51.3)	37	159	19 (12.0)	11 (6.9)	30 (18.9)	129	**< 0.0001**
Familial BC and OC^b^	26	21 (80.8)	0 (0)	21 (80.8)	5	25	7 (28.0)	1 (4.0)	8 (32.0)	17	**0.0005**
Age at diagnosis of familial BC/OC (years)											
≤ 30	41	24 (58.5)	2 (4.9)	26 (63.4)	15	25	11 (44.0)	0 (0)	11 (44.0)	14	NS
31–40	32	22 (68.8)	0 (0)	22 (68.8)	10	59	9 (15.2)	6 (10.2)	15 (25.4)	44	**< 0.0001**
41–50	20	11 (55.0)	0 (0)	11 (55.0)	9	71	5 (7.0)	3 (4.2)	8 (11.3)	63	**< 0.0001**
> 50	9	1 (11.1)	0 (0)	1 (11.1)	8	29	1 (3.4)	3 (10.3)	4 (13.8)	25	NS
Early-onset BC (regardless of a family history of BC/OC)											
≤ 30 years	131	37 (28.2)	2 (1.5)	39 (29.8)	92	172	19 (11.0)	6 (3.5)	25 (14.5)	147	**0.0003**
Ethnicity											
Punjabi	151	56 (37.1)	2 (1.3)	58 (38.4)	93	229	28 (12.2)	9 (3.9)	37 (16.2)	192	**< 0.0001**
Pathan	19	6 (31.6)	0 (0)	6 (31.6)	13	54	3 (5.6)	4 (7.4)	7 (13.0)	47	**0.01**
Others^c^	20	8 (40.0)	0 (0)	8 (40.0)	12	48	3 (6.2)	5 (10.4)	8 (16.7)	40	**0.003**
Unknown	2	1 (50.0)	0 (0)	1 (50)	1	0	0	0	0	0	

*P* values marked in bold are statistically significant

*BC* breast cancer, *NS* non-significant, *OC* ovarian cancer, *TNBC* triple-negative breast cancer

^a^Fisher’s exact test; *BRCA1* carriers *vs.* non-carriers. ^b^Only female index cases affected with BC were included. ^c^Other: minor ethnic groups including Urdu speaking, Saraiki, Kashmiri, Balochi, Indian migratory, Sindhi, Gujrati, Persian speaking, mixed/multiracial

In this study, age appeared to have a marked influence on the *BRCA1* mutation frequency in familial breast/ovarian cancer patients diagnosed with TNBC. In patients over 50 years of age, the frequency was 11.1 %. For younger patients the frequency was 58.5 % for those ≤ age 30, 68.8 % for those between 31 and 40 years, and 55 % for those between 41 and 50 years. The *BRCA1* mutation frequency in the age subgroups 31–40 and 41–50 years were higher in patients with TNBC than those with non-TNBC (68.8 % *vs.* 15.2 %, *P* < 0.0001 and 55 % *vs.* 7 %, *P* < 0.0001). Higher *BRCA1* mutation frequency was observed in early-onset breast cancer patients regardless of family history of breast/ovarian cancer (28.2 % *vs.* 11 %, *P =* 0.0003).

In this study, analysis by ethnicity showed that *BRCA1* mutation frequency in patients with TNBC belonging to the ethnic group of Punjabis, Pathans and other minor ethnic groups was higher than observed in non-TNBC patients (37.1 % *vs.* 12.2 %, *P* < 0.0001; 31.6 % *vs.* 5.6 %, *P* = 0.01 and 40 % *vs.* 6.2 %, *P =* 0.003), respectively.

### Results from logistic regression analysis

*BRCA1/2* mutation carriers and non-carriers were diagnosed with breast cancer at similar age. Each additional year at diagnosis translated into a 1 % lower risk of carrying *BRCA1* mutations and a 1 % higher risk of harboring *BRCA2* mutations, but differences did not reach statistical significance (Ratio of the probability of carrying *BRCA1/2* mutations (RP) = 0.99; 95 % CI 0.97–1.01; *P* = 0.34 and RP = 1.01; 95 % CI 0.97–1.05; *P* = 0.56), respectively (Table 4). Patients with a family history of breast cancer, and in particular patients with a family history of breast and ovarian cancer, showed 237 and 1172 % increased risk of carrying *BRCA1* mutations, respectively, compared to women affected by early-onset breast cancer (Global *P* < 0.0001) (corresponding RP = 3.37; 95 % CI 1.96–5.80; and RP = 12.72; 95 % CI 6.22–26.0, respectively). Patients presenting with breast tumor histology of other than invasive ductal carcinoma showed a 73 % decreased risk of *BRCA1* mutations (RP = 0.27; 95 % CI 0.08–0.89; *P* = 0.03). The prevalence of *BRCA1* mutations also varied with tumor grade; women affected by grade 3 tumors showed the highest risk of carrying a *BRCA1* mutation (Global *P* < 0.0001). In comparison with patients diagnosed with non-TNBC, patients affected by TNBC showed a 390 % higher risk of *BRCA1* mutations (RP = 4.90; 95 % CI 3.09–7.77; *P* < 0.0001).

**Table 4:** Ratio of the probability of carrying *BRCA1/2* mutations in the investigated patients collective based on univariate logistic regression models

		Non-carriers		*BRCA1* mutation carriers					*BRCA2* mutation carriers				
Variables	Level	*n*	%	*n*	%	RP	95 % CI	*P*	*n*	%	RP	95 % CI	*P*
Age^a^	Cont.	398	100	105	100	0.99	0.97 to 1.01	0.34	20	100	1.01	0.97 to 1.05	0.56
Family phenotype	1 early onset BC (≤ 30 years)	210	53	21	20	Ref.		**< 0.0001**	6	30	Ref.		0.13
	Familial BC	166	42	56	53	3.37	1.96 to 5.80		13	65	2.74	1.02 to 7.37	
	Familial BC and OC	22	6	28	27	12.72	6.22 to 26.0		1	5	1.59	0.18 to 13.82	
Menopausal status	Postmenopausal	68	17	10	10	0.51	0.25 to 1.03	0.17	3	15	0.85	0.24 to 3.00	0.97
	Premenopausal	329	83	95	90	Ref.			17	85	Ref.		
	Unknown	1	0	0	0	-			0	0	-		
Ethnicity	Other	53	13	12	11	0.77	0.39 to 1.50	0.17	5	25	2.45	0.82 to 7.32	0.25
	Pathan	60	15	9	9	0.51	0.24 to 1.07		4	20	1.72	0.53 to 0.53	
	Punjabi	285	72	84	80	Ref.			11	55	Ref.		
Histology	Ductal	359	90	102	97	Ref.		**0.03**	18	90	Ref.		0.98
	Other	39	10	3	3	0.27	0.08 to 0.89		2	10	1.02	0.23 to 4.57	
Tumor size	Unknown	80	20	27	26	1.30	0.76 to 2.23	0.73	4	20	0.91	0.28 to 2.97	0.99
	pT1	78	20	19	18	0.94	0.52 to 1.70		4	20	0.93	0.28 to 3.05	
	pT2	181	45	47	45	Ref.			10	50	Ref.		
	pT3	55	14	11	10	0.77	0.37 to 1.59		2	10	0.66	0.14 to 3.09	
	pT4	4	1	1	1	0.96	0.11 to 8.82		0	0	-		
Tumor grade	1	5	1	0	0	-		**< 0.0001**	0	0	-		0.16
	2	116	29	6	6	0.14	0.06 to 0.33		9	45	2.45	0.92 to 6.52	
	3	253	64	94	90	Ref.			8	40	Ref.		
	Unknown	24	6	5	5	0.56	0.21 to 1.51		3	15	3.95	0.98 to 15.9	
Lymph node status	Negative	146	37	47	45	1.49	0.95 to 2.34	0.13	6	30	0.73	0.27 to 1.97	0.83
	Positive	232	58	50	48	Ref.			13	65	Ref.		
	Unknown	20	5	8	8	1.86	0.77 to 4.45		1	5	0.89	0.11 to 7.18	
TNBC status	Non-TNBC	279	70	34	32	Ref.		**< 0.0001**	18	90	Ref.		0.07
	TNBC	119	30	71	68	4.90	3.09 to 7.77		2	10	0.26	0.06 to 1.14	

*P* values marked in bold are statistically significant

*BC* breast cancer, *OC* ovarian cancer, *Ref.* reference, *RP* ratio of the probability of carrying *BRCA1/2* mutations

^a^Median age (5^th^ and 95^th^ percentiles) were 30 years (23 to 54) for non-carriers, 30 years (24 to 48) for *BRCA1*, and 32 years (24 to 53) for *BRCA2* mutation carriers

The association between TNBC and prevalent *BRCA1* mutations was independent of the simultaneous consideration of family history, tumor histology and tumor grade in a multiple logistic regression model (Table 5). After adjustment for family history, tumor histology and tumor grade, patients affected by TNBC showed a 323 % higher risk of *BRCA1* mutations than non-TNBC patients (RP = 4.23; 95 % CI 2.50–7.14; *P* < 0.0001).

**Table 5:** Ratio of the probability of carrying *BRCA1/2* mutations in the investigated patients collective relying on multiple logistic regression model

		Non-carriers		*BRCA1* mutation carriers				
Variables	Level	*n*	%	*n*	%	RP	95%CI	*P*
Family phenotype	1 early onset BC (≤ 30 years)	210	53	21	20	Ref.		**< 0.0001**
	Familial BC	166	42	56	53	4.98	2.77 to 8.97	
	Familial BC and OC	22	6	28	27	16.22	7.22 to 36.5	
Histology	Ductal	359	90	102	97	Ref.		0.36
	Other	39	10	3	3	0.53	0.13 to 2.08	
Tumor grade	1	5	1	0	0	-		**0.001**
	2	116	29	6	6	0.17	0.07 to 0.43	
	3	253	64	94	90	Ref.		
	Unknown	24	6	5	5	0.39	0.12 to 1.20	
TNBC status	Non-TNBC	279	70	34	32	Ref.		**< 0.0001**
	TNBC	119	30	71	68	4.23	2.50 to 7.14	

*P* values marked in bold are statistically significant

*BC* breast cancer, *OC* ovarian cancer, *Ref.* reference, *RP* ratio of the probability of carrying *BRCA1/2* mutations

## Discussion

We report a comprehensive analysis of the prevalence of *BRCA1* and *BRCA2* germline mutations in Pakistani patients with TNBC and non-TNBC selected for age of onset or family history of breast/ovarian cancer. Results from our analysis showed that 97 % of all *BRCA1/2* mutations in patients with TNBC were found in the *BRCA1* gene. The *BRCA1* mutation frequency in patients diagnosed at early age who did not report a family history of breast/ovarian cancer, patients diagnosed at early age irrespective of family history, patients with a family history of breast cancer, and patients with a family history of breast and ovarian cancer were approximately 3 to 4 times higher than those observed in non-TNBC patients. The diagnosis of TNBC independently increased the risk of carrying a *BRCA1/2* mutation. Several studies have demonstrated the relevance of TNBC status as a criterion for genetic *BRCA* testing [7, 13, 15, 22, 26–28]; our study confirms this observation for patients with TNBC in an Asian population from Pakistan.

Pakistani women were diagnosed with TNBC at a younger age and with higher grade tumors than non-TNBC. These findings confirm those from previous studies conducted among Asian [4, 29], North-American [30, 31] and African-American patients [31, 32]. A lower rate of lymph node involvement was observed in Pakistani patients with TNBC than non-TNBC, which is in line with previous data from other Asian [29, 33, 34] and North-American studies [35]. A higher rate was observed in one study among North-Americans [30], while no difference was observed in a European study [36]. The discrepant data may be explained by differences in the study design or the IHC cut-off values for ER and PR negativity. While data from the study of Dent and colleagues were based on unselected cases and a cut-off for ER/PR negativity of < 10 % of tumor cells staining positive, the Pakistani study participants were selected for young age or family history of breast/ovarian cancer and the threshold for negative ER/PR result was < 1 % of tumor cells staining positive.

In Pakistan, 42 distinct mutations including 40 in *BRCA1* and two in *BRCA2* were identified in patients with TNBC. Of these mutations, 17 mutations (including five mutations previously identified in Pakistani breast/ovarian cancer patients) are population-specific as they were not identified in other populations [23, 37]. Twenty-five recurrent *BRCA1/2* mutations (including 18 mutations previously reported in Pakistani breast/ovarian cancer patients) have also been described elsewhere in the world, indicating that majority of mutations found in the current study did not differ from those previously reported in Pakistan or elsewhere.

In most Western studies, the mean age of diagnosis of TNBC in *BRCA1* mutation carriers was significantly lower than in non-carriers [7, 13, 15]. No difference in the age of TNBC diagnosis between *BRCA1* carriers and non-carriers was detected in some studies on early-onset or familial cases from the US [6] and Singapore [18]. In contrast, Pakistani *BRCA1* carriers were two years older at TNBC diagnosis than non-carriers implying that other environmental or genetic factors may be operant in TNBC in this group of women. It is also possible that the diverse results are due to differences in study design, selection criteria or ethnicity.

Pakistani women are usually diagnosed with breast cancer below 40 years of age [38] and often present with advanced disease [39]. In the current study, *BRCA1* mutations were identified in 14.4 % of early-onset patients with TNBC, who had no family history of breast/ovarian cancer. Lower *BRCA1* mutations frequencies of 4.3, 7.4 and 8.7 % were observed in other studies conducted in China, Italy, and the UK, respectively [9, 22, 40]. However, given the small number of patients with TNBC diagnosed < 30 years of age investigated in these studies (*n* = ≤ 30), the percentages may not be truly representative.

In the present study *BRCA1/2* mutations were identified in 58.8 % of patients with TNBC, who reported a family history of breast/ovarian cancer. In other Asian studies performed in China, Malaysia, and Korea and also in Caucasian studies conducted in Australia, Europe, and the United States, the mutation frequencies were similar or lower ranging from 20.8 to 59.5 % [17, 22, 26, 41, 42] and 11.6 to 62 % [7, 8, 10–13, 15], respectively. The varying mutation frequencies obtained in these studies may be explained by differences in sample size, mutation detection assays used, or ethnic origin of study participants.

The low frequency of *BRCA2* mutations detected in our study is in keeping with prior reports and suggests that *BRCA2* may not play an important role in the development of early-onset TNBC. With the exception of one small German study that included 30 patients with TNBC [11], *BRCA2* mutations were less common than *BRCA1* mutations in several studies among patients of European or North-American origin [9, 14, 15, 28] and patients from Asia [22, 26] including the present one. These data indicate the tendency for *BRCA1* carriers to primarily develop TNBC compared to *BRCA2* carriers, which most commonly develop ER positive breast tumors [43].

Recommendations for genetic *BRCA1/2* testing for patients with TNBC are not universally accepted and vary between professional societies [13] and studies [15, 22, 26, 28]. The National Comprehensive Cancer Network (NCCN) guidelines recommend that women with TNBC diagnosed before or at age 60 should be considered for genetic *BRCA1/2* testing (NCCN Guidelines), while the guidelines of the European Society of Medical Oncology [44] and the Cancer Institute New South Wales (https://www.eviq.org.au) recommend testing if TNBC is diagnosed under the ages of 50 and 40 years, respectively. Moreover, testing was suggested to Mexican patients affected by disease below age 60 [28], below or at age 50 to patients from the UK [27], China [22] and Malaysia [26], and irrespective of age to Polish and Australian patients [15]. The high frequency of *BRCA1* mutations in Pakistani patients with a family history of breast/ovarian cancer diagnosed with TNBC below or at age 50 and in early-onset patients diagnosed before or at age 30 irrespective of family history suggest that genetic testing should be considered for these groups of women. Testing women with TNBC diagnosed below age 50 has previously been shown to be a cost-effective strategy [45]. Given the financial burden these considerations are of particular importance for developing countries like Pakistan.

Recently, deleterious mutations in 14 known breast cancer susceptibility genes including *BRCA1, BRCA2*, and *RAD51C* were identified at a frequency of 3.7 % in a large series of 1,824 patients with TNBC unselected for family history of breast cancer [7]. As in the study reported by Couch and colleagues*,* no mutations in the *CHEK2* and *TP53* genes were observed in two Pakistani studies among 374 (including 103 with TNBC) [46] and 105 (including 47 with TNBC) breast/ovarian cancer patients [47], respectively. Recently, a deleterious mutation (c.5101C > T) in the *FANCM* gene was identified in *BRCA1/2*-negative familial patients with TNBC from Finland [48]. This mutation was not detected in a Pakistani study that included 117 patients with TNBC [49].

There are several limitations of our study. First, we have screened only patients with TNBC, who were selected for early-age of onset (≤ 30 years) or family history of breast/ovarian cancer. Hence the selection of high-risk patients may explain the higher *BRCA1/2* mutation frequency observed in our study compared to those that evaluated unselected TNBC patients. Secondly, we did not use *BRCA1/2* prediction models. However, given the previously observed inaccuracy of these algorithms in predicting risk precisely in Asian populations, limits the usefulness of these algorithms and warrants further investigation [50, 51]. Strengths of the present study include the sample size (*N* = 523) comprising sufficiently larger number of early-onset breast cancer (≤ 30 years) women (*n* = 303) with TNBC (*n* = 131) or non-TNBC (*n* = 172) compared to studies reported from Asia previously. Additionally, our study evaluated the complete coding regions of the *BRCA1* and *BRCA2* genes that were comprehensively screened for both, small-range mutations and large genomic rearrangements. Screening for both types of mutations has only been reported in few studies performed previously [10, 26]. Yet another strength was that all data were generated at a single institution, therefore no variability was introduced by using different methods for tumor grading and IHC analysis and evaluation and the pathologist, who evaluated the ER, PR, and HER2 status, was blinded to the mutation status. Finally, the majority of study participants (73.4 %) were recruited within one year of disease presentation, which minimizes the likelihood of survival bias.

## Conclusions

We found high prevalence and predominance of *BRCA1* germline mutations in Pakistani women with TNBC compared to patients with non-TNBC presenting before or at age 30 irrespective of family history of breast/ovarian cancer and before or at age 50 with familial breast cancer or familial breast and ovarian cancer. The association between TNBC status and presence of *BRCA1* mutations was independent of the simultaneous consideration of family phenotype, tumor histology, and tumor grade in a multiple logistic regression model. Our data suggest that TNBC status should be incorporated as a criterion for genetic *BRCA1* testing in Pakistan. Identification of individuals with *BRCA1* germline mutations will enable physicians to optimize cancer management for this high risk phenotype.

## Additional files

Additional file 1: Supplementary methods *BRCA* mutation analyses. (DOCX 17 kb) Additional file 2: Table S1. Deleterious *BRCA1/2* germline mutations in Pakistani patients with TNBC. (DOCX 24 kb)

## Abbreviations

DHPLC: Denaturing High-Performance Liquid Chromatography
ER: Estrogen Receptor
FFPE: Formalin-Fixed Paraffin-Embedded
HER2: Human Epidermal Growth Factor Receptor 2
IHC: Immunohistochemical
MLPA: multiplex ligation-dependent probe amplification
NCCN: National Comprehensive Cancer Network
PR: Progesterone Receptor
SKMCH & RC: Shaukat Khanum Memorial Cancer Hospital and Research Centre, Lahore, Pakistan
TNBC: Triple-Negative Breast Cancer
