# Supplementary material for: High prevalence and predominance of BRCA1 germline mutations in Pakistani triple-negative breast cancer patients
Source: BMC Cancer. 2016 Aug 23;16(1):673. doi: 10.1186/s12885-016-2698-y (PMC4995655; doi:10.1186/s12885-016-2698-y)
Supplement: Additional file 1: — Supplementary methods BRCA mutation analyses. (DOCX 17 kb) [file 12885_2016_2698_MOESM1_ESM.docx]

**Additional file 1**

*BRCA mutation analyses*

**SSCP analysis**

Amplified PCR product of patient samples were diluted 1:10 in formamide buffer (98% formamide, 10 mmol/l EDTA, pH 8, 0.025% bromophenol blue, 0.025% xylene cyanol), heated to 95°C for five minutes and chilled on ice for five minutes. Three μl of this mixture were loaded onto vertical non-denaturing 6% polyacrylamide gels and electrophoresed at 6 W constant power for 7-16 hours in 0.6×TBE buffer at 4°C. Furthermore, samples were electrophoresed on non-denaturing 6% polyacrylamide gels containing 5% glycerol at room temperature. Variant bands were detected by autoradiography.

**PTT analysis**

*BRCA1* exon 11, *BRCA2* exons 10 and 11 were screened by PTT assay. *BRCA1* exon 11 was amplified in 3 overlapping fragments, *BRCA2* exon 11 in 3 to 5 overlapping fragments and *BRCA2* exon 10 in a single fragment. PCR amplification of the segments was as follows: 50 ng genomic DNA was used as template in a 25 μl reaction volume using standard PCR conditions except that the primer concentration was 0.5 μM. Samples were amplified using the following cycling conditions: 95°C for 12 minute, 94°C for 30 seconds, 53°C (for *BRCA1*) or 52°C (for *BRCA2*) for 30 seconds, 72°C for two minutes, 35 times. Coupled transcription and translation was done using 1-3 μl of PCR product in a total volume of 7.5 μl which included 3 μl of TNT T7 reticulocyte lysate (Promega, Madison) and 0.5 μl of ^35^S-cysteine (ICN, Irvine). Labelled protein products were denatured and size fractionated on 12% SDS-PAGE, dried, and autoradiographed.

**DHPLC analysis**

DHPLC analysis was performed using the WAVE system (Transgenomics, Omaha, NE). The PCR products were subjected to heteroduplex formation by denaturing at 95°C for 5 minute, followed by gradual reannealing to 25°C over 25 minutes. The PCR products were loaded to the preheated DNASep column (Transgenomics, Omaha, NE) with homoduplexes and heteroduplexes eluting to it differentially under denaturing conditions. The samples were eluted at a flow rate of 0.9 ml/min using a linear gradient of acetonitrile in 0.1 M triethylamine acetate buffer. The starting gradient varied among amplicons, depending on the DNA sequence and fragment size. The analysis was carried out at 2 to 3 temperatures for amplicons containing several melting domains. Eluted DNA fragments were detected by a UV detector (Transgenomics, Omaha, NE).

**DNA sequence analysis**

Each sample revealing variants detected by either SSCP, DHPLC or PTT analysis was directly sequenced using BigDye Terminator v.3 Cycle Sequencing Kit (Applied Biosystems, California, USA). The sequencing reaction was purified with BigDye XTerminator Purification Kit (Applied Biosystems, California, USA) and sequenced on an automated 3500 Genetic Analyzer (Applied Biosystems, California, USA) according to the manufacturer’s instructions. Bidirectional genomic DNA sequencing was performed to confirm the presence of a mutation. All mutations were reconfirmed on a second blood sample.
