# Supplementary material for: High prevalence and predominance of BRCA1 germline mutations in Pakistani triple-negative breast cancer patients
Source: BMC Cancer. 2016 Aug 23;16(1):673. doi: 10.1186/s12885-016-2698-y (PMC4995655; doi:10.1186/s12885-016-2698-y)
Supplement: Additional file 2: Table S1. — Deleterious BRCA1/2 germline mutations in Pakistani patients with TNBC. (DOCX 24 kb) [file 12885_2016_2698_MOESM2_ESM.docx]

**Table S1** Deleterious *BRCA1/2* germline mutations in Pakistani TNBC patients

| **Family** | **Exon** | **HGVS designation** | | **Mutation type^b^** | **Reported in BIC (No. of entries^c^)** |
| --- | --- | --- | --- | --- | --- |
|  |  | **Nucleotide change^a^** | **Effect on protein** |  |  |
| ***BRCA1* mutations** |  |  |  |  |  |
| 89, 147, 229, 314, 379, 498 | 1-2 deletion |  |  | LGR | Yes (42^d^) |
| 62 | 2 | c.66dup | p.(Glu23Argfs*18) | FS | Yes (32^e^) |
| 73 | 2 | c.68_69del | p.(Glu23Valfs*17) | FS | Yes (2036^e^) |
| 85 | Intron 4 | c.135-1G>T | Splicing site | SP | Yes (30^e^) |
| 254 | 7 | c.335del | p.(Asn112Ilefs*7) | FS | No |
| 1 | 7 | c.431del | p.(Asn144Ilefs*19) | FS | No^e^ |
| 159 | 8 | c.470_471del | p.(Ser157*) | FS | Yes (8^e^) |
| 340, 626 | 11 | c.685del | p.(Ser229Leufs*5) | FS | Yes (2) |
| 470 | 11 | c.784C>T | p.(Gln262*) | NS | No |
| 669 | 11 | c.1008del | p.(Glu337Lysfs*4) | FS | No^e^ |
| 241 | 11 | c.1190del | p.(Asp397Alafs*13) | FS | No |
| 336 | 11 | c.1471C>T | p.(Gln491*) | NS | Yes (4) |
| 328, 557 | 11 | c.1793T>G | p.(Leu598*) | NS | Yes (1^e^) |
| 574 | 11 | c.1961dup | p.(Tyr655Valfs*18) | FS | Yes (13^e^) |
| 488 | 11 | c.2149G>T | p.(Glu717*) | NS | No |
| 35, 236, 283, 489, 493 | 11 | c.2269del | p.(Val757Phefs*8) | FS | Yes (10^e^) |
| 421, 510, 619 | 11 | c.2405_2406del | p.(Val802Glufs*7) | FS | Yes (5^e^) |
| 415, 660 | 11 | c.2603C>G | p.(Ser868*) | NS | Yes (11^e^) |
| 411 | 11 | c.2971A>T | p.(Lys991*) | NS | No |
| 247 | 11 | c.2981_2982del | p.(Cys994*) | FS | No |
| N13, 399 | 11 | c.3339_3341del | p.(Tyr1113*) | FS | No |
| 279, 445 | 11 | c.3598C>T | p.(Gln1200*) | NS | Yes (21^e^) |
| 210, 211, 313, 332, 652 | 11 | c.3770_3771del | p.(Glu1257Glyfs*9) | FS | Yes (23^e^) |
| N4 | 11 | c.4065_4068del | p.(Asn1355Lysfs*10) | FS | Yes (144^e^) |
| 57, 318 | 12 | c.4183C>T | p.(Gln1395*) | NS | Yes (28^e^) |
| 408 | 13 | c.4327C>T | p.(Arg1443*) | NS | Yes (128) |
| 523, 598 | Intron 14 | c.4485-1G>A | Splicing site | SP | Yes (2^e^) |
| 11, 79, 139, 220, 275, 512 | 15 | c.4508C>A | p.(Ser1503*) | NS | Yes (1^e^) |
| 609 | 15 | c.4665del | p.(Arg1555Serfs*4) | FS | No |
| 611 | 16 | c.4862del | p.(Asp1621Valfs*12) | FS | No |
| 21 | 17 | c.5030_5033del | p.(Thr1677Ilefs*2) | FS | Yes (18^e^) |
| 249 | 17 | c.5035del | p.(Leu1679*) | FS | Yes (2) |
| 276 | Intron 17 | c.5074+1G>A | Splicing site | SP | Yes (3) |
| 121 | 20 | c.5257dup | p.(Arg1753Lysfs*77) | FS | No^e^ |
| 187 | 20-21 deletion |  |  | LGR | No |
| 181 | Intron 20 | c.5278-1G>C | Splicing site | SP | No^e^ |
| 193, 261 | 21-24 deletion |  |  | LGR | Yes (7^d^) |
| 338 | 22 | c.5361_5362del | p.(Cys1787Trpfs*42) | FS | No |
| 248 | Intron 23 | c.5468-2A>T | Splicing site | SP | No |
| 18, 48, 329, 377 | 24 | c.5503C>T | p.(Arg1835*) | NS | Yes (74^e^) |
| ***BRCA2* mutations** |  |  |  |  |  |
| 58 | 11 | c.2990T>G | p.(Leu997*) | NS | No^e^ |
| 295 | 11 | c.5722_5723del | p.(Leu1908Argfs*2) | FS | Yes (43^e^) |

^a^ Numbering starts at the first A of the first coding ATG (located in exon 2) of NCBI GenBank accession number U14680 (*BRCA1*) and U43746 (*BRCA2*)

*^b^ FS* frameshift mutation, *LGR* large genomic rearrangement, *MS* missense mutation, *NS* nonsense mutation, *SP* splice site mutation

^c^ *BIC* Breast Cancer Information Core database ( <https://research.nhgri.nih.gov/bic/>), date last accessed June 14, 2016

^d^ Not available in BIC database, however reported in various other studies

^e^ Previously reported in Pakistani breast/ovarian cancer cases [23,37]
